# Effect of Sustained Loading on the Direct Shear Behaviour of Recycled C&D Material–Geosynthetic Interfaces

**DOI:** 10.3390/ma16041722

**Published:** 2023-02-19

**Authors:** Fernanda Bessa Ferreira, Castorina Silva Vieira, Guilherme Mendonça, Maria de Lurdes Lopes

**Affiliations:** CONSTRUCT, Faculty of Engineering, University of Porto, R. Dr. Roberto Frias, 4200-465 Porto, Portugal

**Keywords:** recycled construction and demolition waste, high-strength geotextile, geogrid, large-scale direct shear test, sustained load, long-term interface behaviour, geosynthetic-reinforced system

## Abstract

Recycled construction and demolition (C&D) wastes have been pointed out as a feasible alternative to traditional backfill materials of geosynthetic-reinforced structures, but the current knowledge about the interface behaviour between these unconventional (recycled) materials and the reinforcement is still limited, particularly as far as the time-dependent response is concerned. In this study, a series of large-scale direct shear tests was conducted using an innovative multistage method to evaluate the influence of shear creep loading on the direct shear response of the interfaces between a fine-grained C&D material and two different geosynthetic reinforcements (high-strength geotextile and geogrid). The peak and large-displacement interface shear strength parameters obtained from tests involving sustained loading were compared with those from conventional interface tests. Test results have shown that the shear creep deformation of the interfaces increased with the magnitude of sustained loading. The test specimens experienced additional vertical contraction during the creep stage, which tended to increase with the applied normal stress. For the recycled C&D material–geotextile interface, the sustained loading induced a reduction in the apparent cohesion and a slight increase in the friction angle, when compared to the values estimated from conventional tests. In turn, for the geogrid interface, the apparent cohesion values increased, whereas the friction angle did not significantly change upon shear creep loading.

## 1. Introduction

In 2015, the European Commission (EC) launched the first European Union Circular Economy Action Plan to stimulate Europe’s transition towards a circular economy, where resources are used in a more sustainable way and the pressure on the environment is minimised. Construction and demolition (C&D) was recognised as one of the priority sectors in this Circular Economy Package due to the vast amounts of C&D wastes generated across the European Union (over 35% of total waste generation), as well as their high potential for circularity. In fact, there has been increasing evidence that C&D materials can successfully be recycled and reused in a variety of civil engineering applications, such as concrete production [1,2], road and railway infrastructure [3,4,5,6], ground improvement works [7], geosynthetic-reinforced structures [5,8,9,10,11,12], among others. Taking into account that the construction sector is among the main contributors to the natural resource consumption (accounting for ≈50% of all extracted materials), the incorporation of recycled materials in the construction industry could save massive amounts of natural resources and play a notable role in achieving climate neutrality. More recently, a new European Union Circular Economy Action Plan for a cleaner and more competitive Europe has been adopted, in which Construction and Buildings (with special reference to C&D materials) is still among the key priority value chains [13].

Geosynthetic-reinforced soil systems, such as walls, slopes and bridge abutments, have proven to be a sustainable, cost-effective and technically viable alternative to traditional earth retaining structures. Among the major advantages from the adoption of geosynthetic-reinforced systems is the possibility of using locally available backfill materials, including cohesive and residual soils or recycled waste products, particularly when high quality granular soils are not readily available. The interaction properties at the interfaces between the backfill and the reinforcement are essential for the safe design and stability analysis of the aforementioned structures, since they greatly affect the overall structure performance. Among the experimental methods suitable for characterising backfill material–geosynthetic interaction, the pullout and direct shear tests are the most typically used. While the pullout test is a valuable method for assessing the anchorage strength of the reinforcement [14,15,16,17,18,19], the direct shear test is the most suitable test method for investigating such interaction in cases where sliding of the backfill material on the geosynthetic surface is susceptible to occur [20,21,22,23,24,25,26].

Although some studies have recently suggested that recycled C&D waste may be used in the construction of geosynthetic-reinforced structures as a sustainable replacement for natural soils or aggregates, the interface behaviour between this alternative backfill and the reinforcement deserves further investigation. The short-term recycled C&D material and geosynthetic interface response under direct shear [9,21,23,27] and pullout loading conditions [9,18] has been examined in recent studies with satisfactory results. These studies showed that the mechanical behaviour of the interfaces between properly compacted recycled C&D materials and geosynthetics is generally comparable to that of the interfaces with conventional backfills. However, during the service life of permanent geosynthetic-reinforced soil systems, time-dependent phenomena (i.e., creep and stress relaxation) are likely to occur, which is why the understanding of the time-dependent response of the geosynthetic and confining material interface is of the utmost importance. Nevertheless, there have been very limited studies on this topic, irrespective of the backfill material type [19,27,28,29,30].

Liu and Martinez [28] investigated the creep response of sand–geomembrane interfaces under direct shear mode using a shear box with plan dimensions of 150 mm × 150 mm. Three different levels of shear stress (30, 50 and 75% of the peak shear strength) were employed, with the sustained loading lasting for 240 min. Similar to the creep behaviour of many materials, the sand–geomembrane interfaces exhibited primary creep followed by secondary creep. This study also showed that the post-creep peak shear strength of the studied interfaces was approximately the same as that obtained from the direct shear tests without creep.

Yang et al. [29] studied the shearing creep characteristics of composite geomembrane–soil interfaces by a series of shear creep tests involving graded (i.e., multistage) loading. When the interface achieved stable deformation under a given shear stress level, the stress level increased. This procedure was repeated until the sample reached creep failure. Additionally, an empirical creep model for the aforementioned interfaces was established, which can predict, with reasonable accuracy, the creep displacement under arbitrary loads in practical engineering. However, the authors assumed that the model has limited applicability, and thus further studies should be performed in the future to establish a unified model for shear creep of the studied interfaces under different influential factors.

Cardile et al. [19] analysed the pullout behaviour of soil–geogrid interfaces subjected to sustained tensile loads using a large-scale pullout test apparatus. By comparing the confined tensile strains of the reinforcement with those obtained by in-air tensile creep test, the authors concluded that the creep reduction factor used to reduce the geosynthetic tensile strength in the limit state design approach might be conservative.

A recent study by Lu et al. [31] examined the short-term creep behaviour of geomembrane–geotextile interfaces under dry conditions by a series of direct shear tests under different shear stress levels (30, 50 and 70% of the peak shear strength). The authors found that the creep displacement at the geomembrane–geotextile interface can be estimated using a power equation of time, whose coefficients are functions of the Young’s modulus of the composite material, time, applied normal and shear stresses, as well as peak shear stress and interface friction angle.

Taking into account the scarcity of studies on the direct shear behaviour of recycled C&D material–geosynthetic interfaces, and considering that, to the best of the authors’ knowledge, no previous study has addressed the effects of shear creep loading on the mechanical response of these interfaces, a series of large-scale direct shear tests was carried out using an innovative multistage procedure involving sustained loading. In a previous related paper, the direct shear response of the interface between a recycled C&D material and a high-strength geotextile during and after a period of shear stress relaxation was evaluated and discussed. It was found that, for the conditions investigated, the effect of stress relaxation on the interface peak and residual shear strength was almost negligible [27]. The main purpose of this current study is to investigate the shear creep and post-creep behaviour of the interfaces between a recycled C&D material and two different geosynthetic reinforcements (a high-strength geotextile and a geogrid) and to determine whether the application of sustained loading can affect their peak and residual shear strength parameters, which are essential for the design and stability analysis of geosynthetic-reinforced structures. In the following sections, the materials and methods are described in detail, and the obtained results are presented and discussed.

## 2. Materials and Methods

### 2.1. Recycled C&D Material

**Figure 1:**
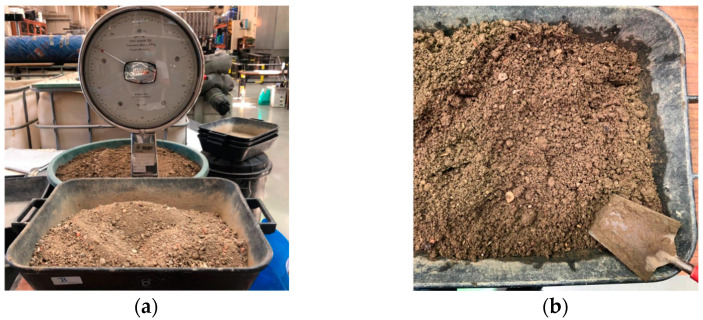
Recycled C&D material: (**a**) initial (air-dried) state; (**b**) at the optimum moisture content.

**Figure 2:**
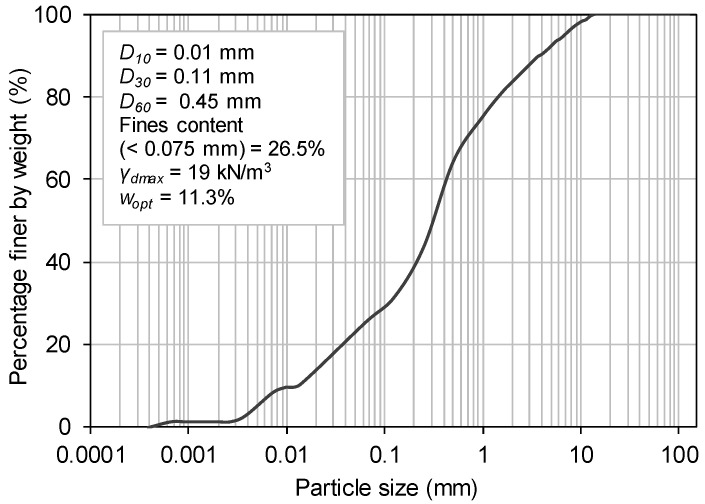
Gradation curve of the recycled C&D material (adapted from [27]).

A fine-grained recycled C&D material was collected from a Portuguese recycling plant and used throughout this study (Figure 1). This fine-grained product resulted from the recycling process of mixed C&D wastes (coming mainly from the maintenance of residential buildings, demolition of masonry fences and recovering of wastes from illegal deposits) and generally receives little market acceptance because of the high percentage of soil and considerable heterogeneity. The particle size distribution of this recycled material is shown in Figure 2, along with some basic geotechnical properties. The particle density (2.53 g/cm^3^) was determined according to the British Standard BS 1377-2:1990 [32]. The optimum compaction parameters (i.e., optimum moisture content, *w_opt_* = 11.3% and maximum dry unit weight, *γ_dmax_* = 19 kN/m^3^) were obtained by the standard Proctor test, as per the European Standard EN 13286-2:2010 [33].

The proportions of the constituents of the C&D material were initially determined on the basis of the European Standard EN 933-11:2011 [34], as shown in Table 1. However, taking into account that, according to this standard, the material passing the 4 mm sieve is not considered, an alternative classification method is proposed to account for the substantial percentage of particles of this particular C&D material falling below 4 mm. Given that the manual sorting of particles passing the 4 mm sieve is virtually impossible, they were listed in Table 1 as “unsorted particles”. Assuming that the unsorted particles consist mainly of soil, this recycled material is mainly composed of soil, with some unbound and hydraulically bound aggregates and concrete/mortar products.

When considering alternative backfill materials for geosynthetic-reinforced structures, such as recycled C&D waste, the assessment of the potential release of dangerous substances resulting in ground water contamination is essential. In this study, the leaching behaviour of the recycled C&D material was evaluated by laboratory leaching tests performed according to the European Standard EN 12457-4:2002 [35]. The concentration of each contaminant and the respective acceptance criteria for inert landfill as per the European Council Decision 2003/33/EC [36] can be found in a previous related publication [27]. It was found that, except for sulphate, whose concentration (1200 mg/kg) did not fulfil the maximum value set by the European legislation for inert landfill (1000 mg/kg), all the pollutants exhibited concentrations falling considerably below the respective threshold values. The noticeable content of sulphate in recycled C&D aggregates is commonly associated with the presence of particles of gypsum drywall [37]. However, according to the provisions of the European legislation [36], if the material does not comply with the limit value for sulphate, it may still be classified as inert material if the leaching does not exceed 6000 mg/kg at a liquid to solid ratio of 10 L/kg.

### 2.2. Geosynthetics

**Figure 3:**
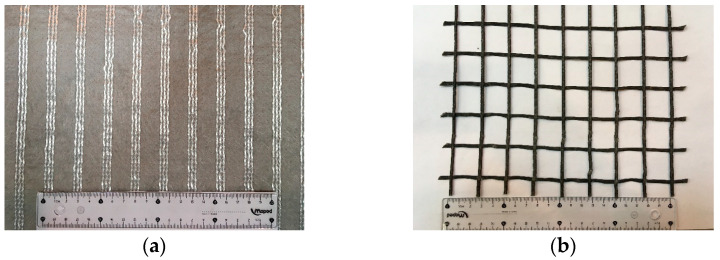
Geosynthetics used: (**a**) high-strength geotextile; (**b**) geogrid.

Two commercially available geosynthetics were used in this study, specifically a uniaxial high-strength geotextile (also termed a geocomposite reinforcement) and a uniaxial woven geogrid with square apertures and mesh size of about 25 mm × 25 mm. The high-strength geotextile (Figure 3a) is composed of high-modulus polyester yarns attached to a continuous filament nonwoven geotextile of polypropylene. Based on the manufacturer’s specifications, the geotextile nominal tensile strength and elongation at nominal strength are 40 kN/m and 10%, respectively. The geogrid (Figure 3b) is manufactured from high modulus polyester yarns, knitted in a flat orientation and covered with a protective polymeric coating. The nominal tensile strength and corresponding elongation as given by the manufacturer are 35 kN/m and 10.5%, respectively.

### 2.3. Direct Shear Test Device and Procedures

**Figure 4:**
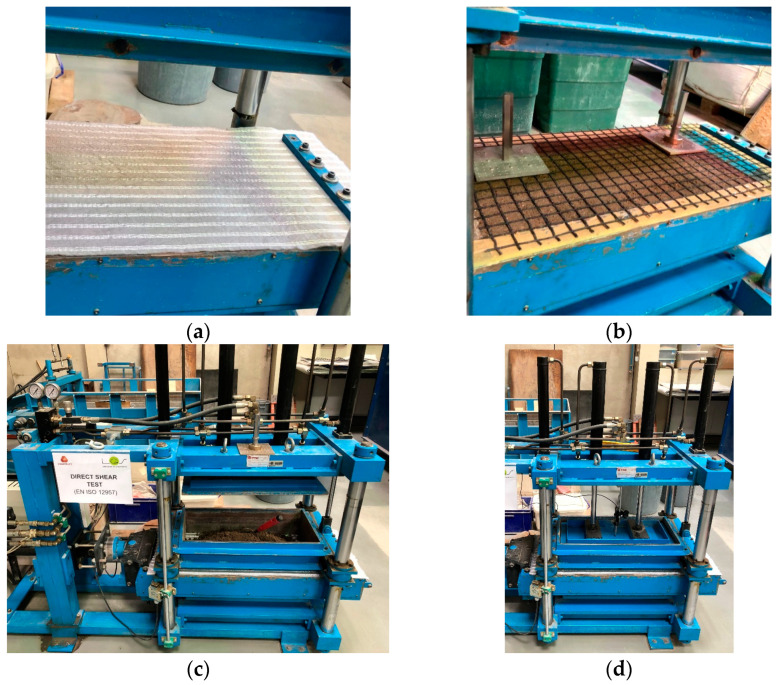
Preparation of test specimens for large-scale direct shear testing: (**a**) geotextile specimen fixed to the lower box (rigid base); (**b**) geogrid specimen fixed to the lower box (filled with recycled C&D material); (**c**) placement of recycled C&D material in the upper box; (**d**) test in progress.

The large-scale prototype direct shear test apparatus used in this experimental research (Figure 4) comprises a shear box, a support structure, a set of servo-hydraulic actuators and associated fluid power unit, an electric cabinet and several displacement and pressure transducers. The lower and upper shear boxes are 800 mm × 340 mm × 100 mm and 600 mm × 300 mm × 150 mm (length × width × thickness), respectively. The tests may be conducted with constant or reduced contact area during shearing. This is accomplished by using either a rigid base or a rigid ring inside the lower box. When the rigid ring is employed, the inner area of the lower box matches that of the upper box (600 mm × 300 mm) and both the lower and upper boxes are filled with soil (or alternative material). The apparatus is capable of performing monotonic and cyclic direct shear tests, as well as short-term creep and stress relaxation tests to investigate the preliminary time-dependent interface behaviour. A full description of this direct shear test apparatus can be found in earlier publications [22,38].

As per the European Standard EN ISO 12957-1:2018 [39], direct shear tests on soil–geosynthetic interfaces shall be performed by fixing the geosynthetic to a rigid, horizontal support installed in the lower shear box, except for geogrids with large apertures (>15 mm) and a relevant percentage of openings (>50% of the overall surface of the specimen), in which case a soil support may alternatively be used. Accordingly, the direct shear tests to characterise the interface involving the high-strength geotextile were performed using a rigid base, over which the geosynthetic specimen was fixed (Figure 4a), whereas the tests on the interface involving the geogrid were performed by filling the lower box with the recycled C&D material (Figure 4b). In this latter case, the C&D material was poured and manually compacted in the lower box using a light compacting hammer, in four individual 25 mm thick layers, up to a total height of 100 mm (compacted thickness). To resemble typical field conditions, the recycled material was compacted at the optimum moisture content (*w_opt_* = 11.3%) and dry unit weight (*γ_d_*) of 17.1 kN/m^3^, corresponding to 90% of the maximum dry density determined from the standard Proctor test, in accordance with the EN 13286-2:2010 [33].

After fixing the geosynthetic specimen to the lower box, the upper box was lowered, leaving a 1 mm gap between its base and the specimen surface. Four layers of recycled C&D material were then placed and compacted in the upper box (Figure 4c), following the same procedures used for the lower box. The normal stress (*σ_n_*) was applied on the top of the recycled C&D material placed in the upper box by a rigid steel plate with pressure-controlled double acting linear actuators and kept constant for 1 h prior to the shearing process (Figure 4d). During the test, the values of normal stress, vertical displacement of the loading plate centre, horizontal displacement of the lower box and shear stress mobilised at the interface were continuously monitored.

### 2.4. Experimental Programme

A series of large-scale direct shear tests was conducted to evaluate the behaviour of the interfaces between the recycled C&D waste and the geosynthetics (geotextile and geogrid) using both conventional and multistage methods. Table 2 summarises the test conditions analysed in this current study. The conventional interface tests were carried out according to EN ISO 12957-1:2018 [39] by applying a constant displacement rate (1 mm/min) until an interface shear deformation of 60 mm was reached.

To investigate the time-dependent mechanical response of the aforementioned interfaces, a novel multistage test method involving sustained shear loading was developed. This multistage procedure consisted of the following three phases: initially, a constant load increment rate (1 kN/min) was imposed until the shear load reached the desired level (*L_C_*); in the second stage (creep stage), the shear load applied at the interface (*L_C_*) was kept constant over a predefined time slot, during which the shear creep displacement was continuously monitored; in the last stage, the test proceeded at a constant displacement rate (1 mm/min) until a maximum horizontal displacement of 60 mm was reached. To investigate the influence of the magnitude of the shear load applied during the creep stage (*L_C_*), different load levels were considered. As indicated in Table 2, these load levels (*L_C_*/*F_max_*) were defined as a proportion of the maximum shear force (*F_max_*) mobilised in the conventional test performed under the same normal stress. The duration of the creep stage (*t_c_*) ranged between 30 and 120 min to evaluate its impact on the interface shear behaviour. The maximum duration of the creep stage was set to 120 min to avoid the overheating of the hydraulic system of the direct shear test apparatus.

The EN ISO 12957-1:2018 [39] recommends the use of normal stresses (*σ_n_*) ranging from 50 to 150 kPa when performing direct shear tests on standard sand–geosynthetic interfaces. In this study, the applied normal stresses ranged from 25 kPa to 150 kPa to cover a wider range of confining stresses that may occur in the field, while also considering the limit values of normal force that may be imposed with the aforementioned direct shear test device. To evaluate the repeatability of results, several tests under *σ_n_* = 100 kPa were carried out twice under identical test conditions.

## 3. Results and Discussion

### 3.1. Conventional Interface Direct Shear Tests

**Figure 5:**
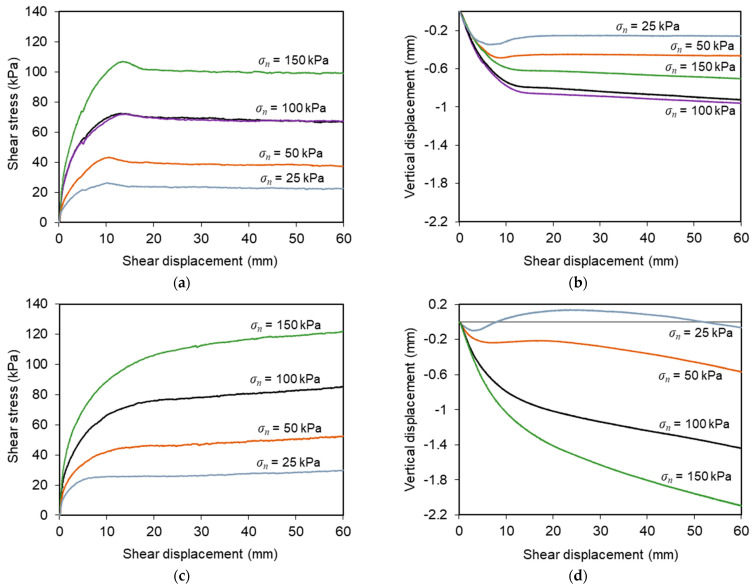
Results from conventional direct shear tests: (**a**) variation of shear stress with shear displacement (recycled C&D material–geotextile interface); (**b**) vertical displacements of the loading plate (recycled C&D material–geotextile interface); (**c**) variation of shear stress with shear displacement (recycled C&D material–geogrid interface); (**d**) vertical displacements of the loading plate (recycled C&D material–geogrid interface).

Figure 5 presents the results from conventional direct shear tests T1–T5 (Figure 5a,b) and T17–T20 (Figure 5c,d) carried out on interfaces with geotextile and geogrid, respectively. As mentioned previously, these tests were performed under normal stresses ranging from 25 to 150 kPa at a constant displacement rate of 1 mm/min.

The shear stress–shear displacement curves associated with the C&D material–geotextile interface (Figure 5a) show well-defined peak shear strengths, whose values increase with the normal stress employed in the tests. Beyond the peak value, the interface shear strength decreased with any further horizontal displacement until a relatively steady state was attained (residual or large-displacement shear strength). It can also be observed that the shear displacement at which the peak shear strength was reached tended to increase with the normal stress.

As shown in Figure 5b, the vertical displacements measured at the loading plate centre revealed essentially a contractile response of the specimens, irrespective of normal stress. The vertical settlement was particularly relevant at the initial stage of the test and tended to stabilise when the strain softening behaviour was completed. Note that the vertical deformation at the end of the shearing process did not exceed 1 mm in these tests.

The evolution of shear stress with shear displacement recorded during the recycled C&D material–geogrid interface tests is shown in Figure 5c. As opposed to the trend exhibited by the interface with the geotextile, strain hardening behaviour was evident, particularly at higher normal stresses, with the shear stress increasing continuously with shear displacement until the end of the test, and no peak of strength was attained.

The vertical deformation of the test specimens during shearing became more pronounced as the normal stress was progressively increased (Figure 5d). In general, only vertical settlement was observed during these tests. The only exception occurred under the lowest normal stress (*σ_n_* = 25 kPa), in which some dilation was identified after the initial phase of vertical contraction. The maximum vertical deformation was about 2.1 mm and took place under the highest normal stress (*σ_n_* = 150 kPa) after a shear displacement of 60 mm (i.e., at the end of the test). This value is considerably higher than that obtained in the direct shear tests involving the geotextile.

The distinct behaviour observed in the direct shear tests represented in Figure 5 is mainly associated with the use of different geosynthetic materials. The open structure of the geogrid enables the mobilisation of the internal strength of the recycled C&D waste within the geogrid apertures, apart from the skin friction along the longitudinal and transverse ribs of the reinforcement during shearing. In contrast, in the direct shear tests of the recycled C&D material–geotextile interface, only the skin friction mechanism contributes to the mobilised interface shear strength and there is no shear plan between recycled C&D waste particles. The differences in the direct shear response of interfaces involving different geosynthetic materials (i.e., geogrid vs. geotextile) were also observed in a previous related study [9].

### 3.2. Effect of Sustained Loading on the Interface Behaviour

#### 3.2.1. Recycled C&D Material–Geotextile Interface

**Figure 6:**
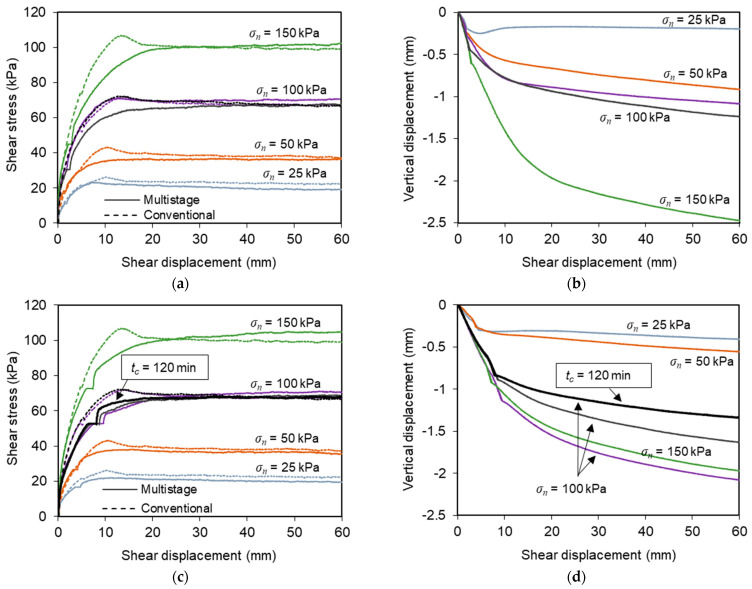
(**a**,**b**) Results from multistage tests on the recycled C&D material–geotextile interface (*L_C_*/*F_max_* = 0.42 and *t_c_* = 30 min); (**c**,**d**) results from multistage tests on the recycled C&D material–geotextile interface (*L_C_*/*F_max_* = 0.62–0.73 and *t_c_* = 30 or 120 min).

Figure 6 presents the results from multistage tests on the C&D material–geotextile interface under sustained loads with distinct characteristics (tests T6–T16). Figure 6a,b is associated with *L_C_*/*F_max_* = 0.42 and *t_c_* = 30 min (tests T6–T10), whereas Figure 6c,d is associated with a higher sustained load level (*L_C_*/*F_max_* = 0.62–0.73) and *t_c_* = 30 or 120 min (tests T11–T16). The shear stress–shear displacement curves from the comparable conventional tests (tests T1–T5) are also superimposed in Figure 6a,c for comparison purposes.

It can be seen from Figure 6a,c that the application of sustained loading led generally to a reduction in the interface maximum shear strength and no peak of strength was obtained, as opposed to the trend identified from the conventional tests. On the other hand, the residual shear strength was not considerably affected by the sustained loading, since the shear strength mobilised after large displacements was rather similar both in the conventional and multistage tests. The curves from the multistage tests also reveal that during the stage in which the shear stress was held constant, some additional shear displacement occurred at the interface, which was attributed to creep.

The influence of the time slot during which the shear load was kept constant (*t_c_*) can be examined from Figure 6c, where the results from the test in which *t_c_* increased to 120 min (T16) are illustrated. Comparing the results from tests T13–14 (*t_c_* = 30 min) and T16 (*t_c_* = 120 min), it appears that increasing the duration of the creep stage resulted in higher interface shear stiffness at the onset of the subsequent shearing phase. However, the maximum and large-displacement shear strengths were not significantly affected.

The vertical deformation of the test specimens recorded from the multistage tests of the C&D material–geotextile interface is illustrated in Figure 6b,d. The deformation consisted essentially of vertical settlement, which tendentially increased with the normal stress. The maximum vertical deformation measured at the end of the tests was about 2.5 mm. In general, the cumulative vertical settlement was greater in the tests involving sustained loading, comparatively to that recorded in the conventional test under the same normal stress (Figure 5b).

**Figure 7:**
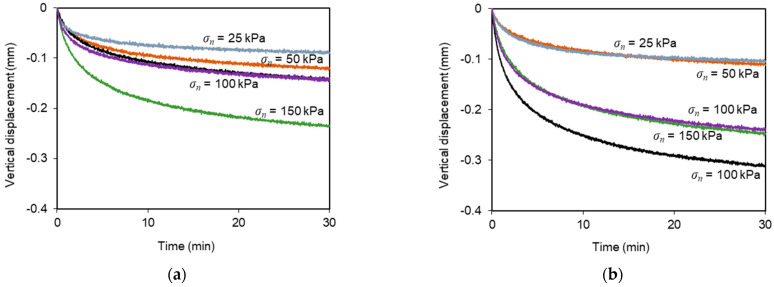
Vertical displacement of the recycled C&D material–geotextile specimen under sustained loading: (**a**) *L_C_*/*F_max_* = 0.42 and *t_c_* = 30 min; (**b**) *L_C_*/*F_max_* = 0.62–0.73 and *t_c_* = 30 min.

Regardless of normal stress or sustained load level, the specimens experienced additional vertical contraction during the creep stage, as shown in Figure 7. This additional deformation was more pronounced at the beginning of the above-mentioned stage and tended to increase with the normal stress value. This type of response was also reported by Liu and Martinez [28] for sand–geomembrane interfaces subjected to shear creep loading.

**Figure 8:**
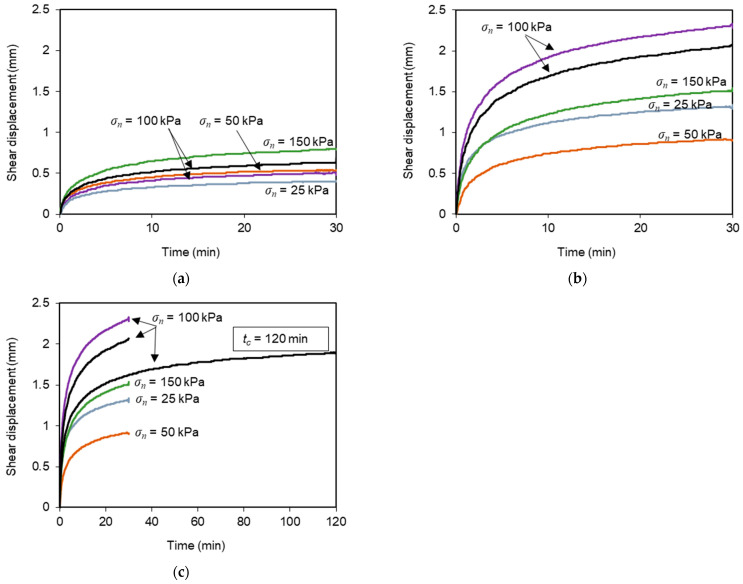
Shear displacement at the recycled C&D material–geotextile interface under sustained loading: (**a**) *L_C_*/*F_max_* = 0.42 and *t_c_* = 30 min; (**b**) *L_C_*/*F_max_* = 0.62–0.73 and *t_c_* = 30 min; (**c**) *L_C_*/*F_max_* = 0.62–0.73 and *t_c_* = 30 or 120 min.

The evolution of shear displacement with elapsed time during the sustained loading stage as obtained from the recycled C&D material–geotextile interface tests (tests T6–T16) is shown in Figure 8. The creep displacement of the interface followed the typical mode of creep deformation of most materials. At the beginning of the shear creep stage, a high initial creep rate was observed, which continuously decreased with time (primary or transient creep), suggesting that the interface experienced an increase in creep resistance or strain hardening (i.e., the deformation became more difficult as the creep deformation increased). Then, a relatively constant creep rate was attained (secondary or steady-state creep). The constancy of creep rate may be explained on the basis of a balance between the competing processes of strain hardening and recovery, the latest being the process by which the ability to experience deformation is retained due to softening [40]. It is noteworthy that, due to limitations of the test apparatus, the creep stage in this study was not long enough to identify the potential occurrence of tertiary creep, which is characterised by an acceleration of creep rate and ultimate failure.

Figure 8 also shows that the creep displacement increased with the magnitude of sustained loading. Indeed, when subjected to a given normal stress, the maximum interface shear creep deformation was considerably higher under *L_C_*/*F_max_* = 0.62–0.73 (Figure 8b), when compared to that under a lower load level, *L_C_*/*F_max_* = 0.42 (Figure 8a). This finding is in agreement with the studies by Liu and Martinez [28] for sand–geomembrane interfaces, Lu et al. [31] for geomembrane–geotextile interfaces and Yang et al. [29] for composite geomembrane–granular material interfaces, in which the creep displacement was larger at higher shear stress levels under the same normal stress. The highest accumulated creep displacement was recorded in test T13 (*L_C_*/*F_max_* = 0.73, *t_c_* = 30 min and *σ_n_* = 100 kPa) and did not exceed 2.3 mm (Figure 8b).

The results from the test in which the sustained loading was applied over a longer duration (i.e., *t_c_* = 120 min) show that, after the initial 30 min of creep loading, the creep rate remained nearly constant and the additional creep displacement measured until the end of the creep stage was almost negligible (Figure 8c). Therefore, the duration of the creep stage employed in most of the multistage tests reported herein (30 min) was deemed sufficient to investigate the preliminary time-dependent interface response.

#### 3.2.2. Recycled C&D Material–Geogrid Interface

**Figure 9:**
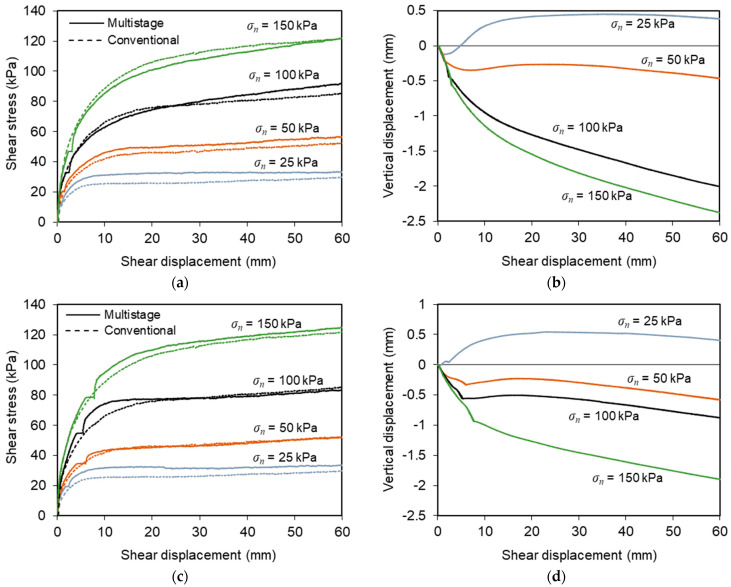
(**a**,**b**) Results from multistage tests on the recycled C&D material–geogrid interface (*L_C_*/*F_max_* = 0.42 and *t_c_* = 30 min); (**c**,**d**) results from multistage tests on the recycled C&D material–geogrid interface (*L_C_*/*F_max_* = 0.70 and *t_c_* = 30 min).

The results from the multistage tests carried out to characterise the influence of sustained loading on the direct shear behaviour of the interface with the geogrid (T21–T28) are plotted in Figure 9.

Figure 9a,c shows the evolution of shear stress with shear displacement from multistage tests involving sustained load levels (*L*_C_/*F_max_*) of 0.42 and 0.70, respectively, as well as the data from the corresponding conventional tests. These results indicate that the overall shear stress–displacement behaviour observed from the multistage tests was comparable to that of the conventional test under the same normal stress. As expected, during the sustained loading stage, an additional shear displacement attributed to creep was mobilised at the interface. Right after the creep stage, the interface stiffness clearly increased. In turn, the large displacement shear strength recorded in the multistage tests was identical to or greater than that measured in the conventional (i.e., benchmark) test.

**Figure 10:**
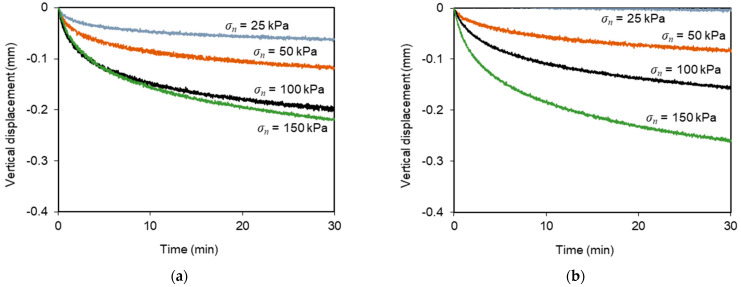
Vertical displacement of the recycled C&D material–geogrid specimens under sustained loading: (**a**) *L_C_*/*F_max_* = 0.42 and *t_c_* = 30 min; (**b**) *L_C_*/*F_max_* = 0.70 and *t_c_* = 30 min.

Figure 9b,d depicts the vertical displacements of the loading plate during these multistage tests. Under the lower normal stress (*σ_n_* = 25 kPa), the specimens underwent considerable dilation. However, under normal stresses ranging from 50–150 kPa, the deformation consisted essentially in vertical contraction throughout the test. The rate of vertical deformation was more significant at the beginning of the shearing process. As previously noted from the test results with the geotextile, during the sustained loading stage, some additional vertical displacements occurred in parallel with the development of shear creep deformation. The data clearly indicate that the vertical displacements mobilised during the creep stage increased progressively with the applied normal stress, irrespective of the sustained load level (Figure 10).

**Figure 11:**
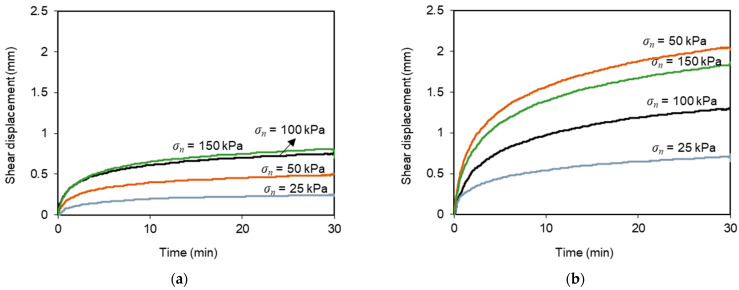
Shear displacement at the recycled C&D material–geogrid interface under sustained loading: (**a**) *L_C_*/*F_max_* = 0.42 and *t_c_* = 30 min; (**b**) *L_C_*/*F_max_* = 0.70 and *t_c_* = 30 min.

The variation of shear displacement during the creep stage, as obtained from the different tests on the recycled C&D material–geogrid interface, is presented in Figure 11a,b for lower and higher sustained load levels, respectively. Similar to what was observed for the interface involving the geotextile, the creep rate was more pronounced in the initial stage of constant shear loading (primary creep), after which the rate of shear displacement accumulation tended to stabilise (secondary creep). From the comparison of the results in Figure 11a,b, it can be concluded that the creep displacement increased with the magnitude of sustained loading, regardless of normal stress. The highest cumulative interface creep displacement was achieved in test T26 (*L_C_*/*F_max_* = 0.70 and *t_c_* = 30 min) and did not exceed 2.0 mm (Figure 11b).

### 3.3. Interface Shear Strength Parameters

**Figure 12:**
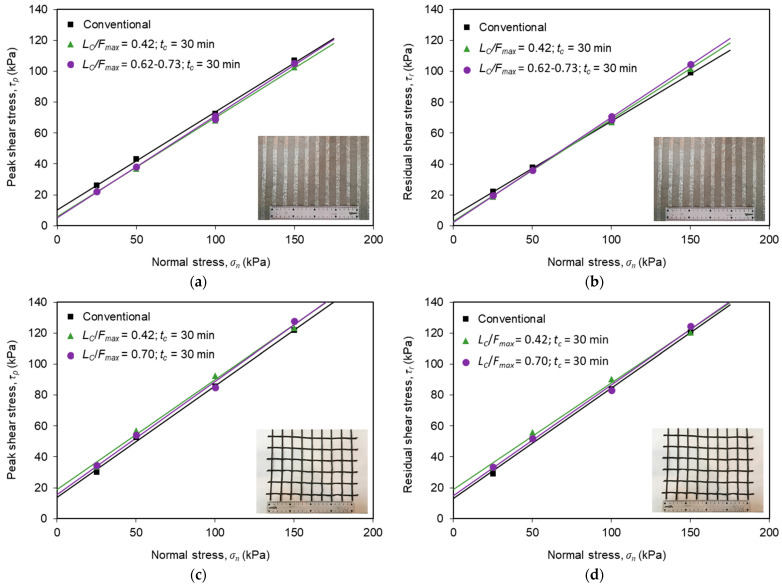
Shear strength envelopes from conventional and multistage tests: (**a**) peak shear strength (recycled C&D material–geotextile interface); (**b**) residual shear strength (recycled C&D material–geotextile interface); (**c**) peak shear strength (recycled C&D material–geogrid interface); (**d**) residual shear strength (recycled C&D material–geogrid interface).

Figure 12 compares the peak and residual shear strength envelopes from conventional and multistage tests on the recycled C&D material–geosynthetic interfaces, which were estimated from the test data by fitting a straight line through the plot of peak or residual shear stress versus the normal stress. Using the Mohr–Coulomb failure criterion, the values of the interface shear strength parameters, specifically the friction angle (*δ*) and apparent cohesion (*c_a_*) were derived (Table 3).

From the analysis of the shear strength envelopes and associated parameters for the recycled C&D material–geotextile interface (Figure 12a,b and Table 3), it becomes apparent that the sustained loading led to a reduction in the apparent interface cohesion (both peak and residual values), in comparison to that derived from tests without sustained loading. This reduction was slightly greater under the higher sustained load level. However, the sustained shear loading did not induce any reduction in the interface peak and residual friction angles. In fact, these parameters slightly increased upon shear creep. Additionally, for the recycled C&D material–geotextile interface, the apparent cohesion values associated with large displacement shear strength were lower than those obtained for peak shear strength.

Concerning the influence of sustained loading on the shear strength parameters of the interface with the geogrid, it can be seen from Figure 12c,d and Table 3 that the apparent cohesion increased, whereas the friction angle did not significantly change upon shear creep loading. Accordingly, the post-creep interface shear strength was approximately the same or even slightly greater that that obtained from the direct shear tests without creep. This finding suggests that the interface shear strength parameters estimated from conventional direct shear tests (current practice) may be considered suitable to characterise the shear strength of this particular interface subjected to short-term creep loading. However, additional tests involving longer durations would be useful for determining whether this conclusion can be generalised for long-term creep loading conditions.

Furthermore, the friction angle and apparent cohesion values estimated from tests with or without sustained loading (Table 3) are well within the range typically reported in the literature for interfaces between geosynthetics and natural soils used as construction material in geotechnical engineering applications. Vieira et al. [38] obtained interface peak friction angle and apparent cohesion values of 31.4° and 7.5 kPa, respectively, for the interface between a silica sand (compacted at its air-dried water content and relative density of 70%) and a high-strength geotextile from conventional direct shear tests using the same large-scale test apparatus. Moreover, Ferreira et al. [22] reported values of interface peak friction angle and apparent cohesion of 36.1° and 1.5 kPa, respectively, for the interface between a Portuguese granite residual soil (tested at *w_opt_* = 11.5% and *γ_d_ =* 17.30 kN/m^3^) and a uniaxial high-strength geotextile, which were also obtained using the same apparatus and identical test procedures. The greater apparent cohesion value obtained in the current study may be justified by the substantially higher content of fine particles (<0.075 mm) of the recycled C&D material (26.5%), in comparison to that of the granite residual soil employed in the aforementioned research (8.0%).

It can also be concluded from Figure 12 and Table 3 that the interface with the geogrid exhibited higher shear strength properties than the geotextile interface under both conventional direct shear and post-shear creep loading conditions. This reflects the beneficial contribution of the recycled C&D material internal strength developed within the geogrid apertures. In fact, it is widely accepted that, when the reinforcement is a geogrid with a significant open area, the mobilisation of the internal soil strength in the geogrid apertures contributes for a high percentage of the overall interface strength under direct shear mode.

## 4. Conclusions

This paper presented the results from a series of large-scale direct shear tests carried out using an innovative multistage method to investigate the shear creep and post-creep behaviour of recycled C&D waste–geosynthetic interfaces. The most relevant conclusions of this study are summarised below.

The application of sustained loading resulted generally in the reduction of the maximum shear strength of the recycled C&D material–geotextile interface. However, for the geogrid interface, the overall shear stress–displacement behaviour observed from the multistage tests was generally similar to that of the corresponding conventional tests. The residual shear strength was not considerably affected by the sustained loading, regardless of geosynthetic type.

During the stage in which the shear stress was held constant, some additional interface shear displacement was visible (i.e., creep deformation). The shear creep displacement of the studied interfaces followed the characteristic mode of creep deformation of most materials. In the initial stage of shear creep loading, a high creep rate was observed which progressively decreased over time (primary creep). Subsequently, a nearly constant creep rate was obtained (secondary creep). The verification of potential occurrence of tertiary creep and eventual interface failure was outside the scope of this study.

For both interfaces, the creep displacement increased with the magnitude of sustained loading. The highest cumulative shear creep displacements recorded at the interfaces with the geotextile and the geogrid after 30 min of creep loading were similar (about 2.3 mm and 2.0 mm, respectively).

The results from the multistage test carried out over a longer duration indicated that, after the initial 30 min of shear creep loading, the creep rate remained nearly constant, and the additional shear creep deformation developed until the end of the creep stage was almost negligible. However, increasing the duration of the creep stage from 30 to 120 min led to higher C&D material–geotextile interface shear stiffness at the onset of the subsequent shearing stage. For the conditions investigated, the influence of creep time on the maximum and large displacement interface shear strengths was not relevant.

Regardless of geosynthetic type, normal stress or sustained load level, the test specimens experienced additional vertical contraction during the shear creep stage (up to 0.3 mm), concurrently with the development of shear creep deformation. This additional vertical deformation was more pronounced at the start of the creep stage and tended to increase with the applied normal stress.

For the recycled C&D material–geotextile interface, the sustained loading induced a reduction in the apparent cohesion, when compared to that estimated from conventional tests. Conversely, the interface friction angle slightly increased. In turn, for the interface with the geogrid, the apparent cohesion increased, whereas the friction angle did not significantly change upon shearing creep. The above trends applied both for peak and residual interface shear strengths.

The interface with the geogrid revealed higher strength properties than the geotextile interface under both conventional direct shear and post-shear creep loading conditions. This is attributed to the positive contribution of the internal shear strength of the C&D material mobilised within the geogrid apertures during shearing.

This paper contributes to a better understanding of the direct shear behaviour of the interfaces between recycled C&D waste and different geosynthetics, which is essential for the design and stability analysis of geosynthetic-reinforced systems built with this unconventional backfill. The interface shear strength parameters derived from the present tests are comparable to the values generally reported in the literature for interfaces between geosynthetics and natural soils. This corroborates that this recycled C&D material could be employed as an alternative sustainable backfill in the construction of geosynthetic-reinforced systems, replacing natural soils or aggregates, which would represent a valuable contribution towards the implementation of circular economy in the construction sector. Additional direct shear tests involving different recycled C&D materials and geosynthetics, as well as longer duration sustained loadings would be useful to gain further insight into the shear creep behaviour of these interfaces under long-term conditions.

## Figures and Tables

**Table 1:** Analysis of the recycled C&D material constituents [27].

Constituents	According to [34] *	Global Value
Concrete, concrete products, mortar, concrete masonry units, *R_c_* (%)	16.0	1.6
Unbound aggregate, natural stone, hydraulically bound aggregate, *R_u_* (%)	45.7	4.6
Clay masonry units, calcium silicate masonry units, aerated non-floating concrete, *R_b_* (%)	3.7	0.4
Bituminous materials, *R_a_* (%)	2.2	0.2
Glass, *R_g_* (%)	0.3	0.0
Other materials, *X* ** (%)	32.1	3.2
Unsorted particles (<4 mm) (%)	-	90
Floating particles, FL (cm^3^/kg)	3.8	-

* Only for particles > 4 mm; ** materials that do not fall into the above categories (e.g., gypsum drywall, cork, non-floating wood and soils resulting from the washing process).

**Table 2:** Summary of conventional and multistage direct shear tests carried out in this study.

Test Number	Geosynthetic Type	Test Method	Normal Stress, *σ_n_* (kPa)	Sustained Shear Load, *L_C_* (kN)	Load Level, *L_C_*/*F_max_*	Time, *t_c_* (min)
T1	Geotextile	Conventional	25	-	-	-
T2	Geotextile	Conventional	50	-	-	-
T3	Geotextile	Conventional	100	-	-	-
T4	Geotextile	Conventional	100	-	-	-
T5	Geotextile	Conventional	150	-	-	-
T6	Geotextile	Multistage	25	2.0	0.42	30
T7	Geotextile	Multistage	50	3.3	0.42	30
T8	Geotextile	Multistage	100	5.5	0.42	30
T9	Geotextile	Multistage	100	5.5	0.42	30
T10	Geotextile	Multistage	150	8.1	0.42	30
T11	Geotextile	Multistage	25	3.4	0.72	30
T12	Geotextile	Multistage	50	4.8	0.62	30
T13	Geotextile	Multistage	100	9.5	0.73	30
T14	Geotextile	Multistage	100	9.5	0.73	30
T15	Geotextile	Multistage	150	13.1	0.68	30
T16	Geotextile	Multistage	100	9.5	0.73	120
T17	Geogrid	Conventional	25	-	-	-
T18	Geogrid	Conventional	50	-	-	-
T19	Geogrid	Conventional	100	-	-	-
T20	Geogrid	Conventional	150	-	-	-
T21	Geogrid	Multistage	25	2.1	0.42	30
T22	Geogrid	Multistage	50	3.7	0.42	30
T23	Geogrid	Multistage	100	5.9	0.42	30
T24	Geogrid	Multistage	150	8.4	0.42	30
T25	Geogrid	Multistage	25	3.5	0.70	30
T26	Geogrid	Multistage	50	6.2	0.70	30
T27	Geogrid	Multistage	100	9.8	0.70	30
T28	Geogrid	Multistage	150	14.0	0.70	30

**Table 3:** Peak and residual shear strength parameters of the recycled C&D material–geosynthetic interfaces.

Interface	Test Method	*L_C_*/*F_max_*	*t_c_* (min)	Peak		Residual	
				*δ* (°)	*c_a_* (kPa)	*δ* (°)	*c_a_* (kPa)
C&D material–geotextile	Conventional	-	-	32.4	10.3	31.4	6.9
	Multistage	0.42	30	32.6	6.0	33.4	2.9
	Multistage	0.62–0.73	30	33.4	5.0	34.2	2.2
C&D material–geogrid	Conventional	-	-	35.8	13.9	35.6	13.0
	Multistage	0.42	30	35.4	19.0	34.6	18.8
	Multistage	0.70	30	36.2	15.8	35.7	14.9

## Data Availability

The data presented in this study are available on request from the corresponding authors. The data are not publicly available due to privacy reasons.
